# Retraction: Exosome-Delivered LncHEIH Promotes Gastric Cancer Progression by Upregulating EZH2 and Stimulating Methylation of the GSDME Promoter

**DOI:** 10.3389/fcell.2022.896056

**Published:** 2022-03-23

The journal and Chief Editors retract the 14 October 2020 article cited above.

Following publication, concerns were raised regarding the integrity of the images in the published figures. The authors failed to provide a satisfactory explanation during the investigation, which was conducted in accordance with Frontiers’ policies. Given the concerns about the images, the editors no longer have confidence in the findings presented in the article.

This retraction was approved by the Chief Editors of Frontiers in Cell and Developmental Biology and the Chief Executive Editor of Frontiers. The authors do not agree to the retraction of this article.

